# MedCPT: Contrastive Pre-trained Transformers with Large-scale PubMed Search Logs for Zero-shot Biomedical Information Retrieval

**Published:** 2023-10-04

**Authors:** Qiao Jin, Won Kim, Qingyu Chen, Donald C. Comeau, Lana Yeganova, W. John Wilbur, Zhiyong Lu

**Affiliations:** 1 National Center for Biotechnology Information (NCBI), National Library of Medicine (NLM), National Institutes of Health (NIH)

## Abstract

**Motivation:**

Information retrieval (IR) is essential in biomedical knowledge acquisition and clinical decision support. While recent progress has shown that language model encoders perform better semantic retrieval, training such models requires abundant query-article annotations that are difficult to obtain in biomedicine. As a result, most biomedical IR systems only conduct lexical matching. In response, we introduce MedCPT, a first-of-its-kind Contrastively Pre-trained Transformer model for zero-shot semantic IR in biomedicine.

**Results:**

To train MedCPT, we collected an unprecedented scale of 255 million user click logs from PubMed. With such data, we use contrastive learning to train a pair of closely-integrated retriever and re-ranker. Experimental results show that MedCPT sets new state-of-the-art performance on six biomedical IR tasks, outperforming various baselines including much larger models such as GPT-3-sized cpt-text-XL. In addition, MedCPT also generates better biomedical article and sentence representations for semantic evaluations. As such, MedCPT can be readily applied to various real-world biomedical IR tasks.

**Availability:**

The MedCPT code and API are available at https://github.com/ncbi/MedCPT.

## Introduction

Information retrieval (IR) is an important step in biomedical knowledge discovery and clinical decision support (Ely, et al., 2005; Gopalakrishnan, et al., 2019). However, most IR systems in biomedicine are keyword-based, which will miss articles that are semantically relevant but have no lexical overlap with the input query. Recent progress in IR and deep learning has shown that dense retrievers, which encode and match queries and documents as dense vectors, can perform better semantic retrieval than traditional sparse (lexical) retrievers such as BM25 (Karpukhin, et al., 2020; Khattab and Zaharia, 2020; Lin, et al., 2022; Nogueira and Cho, 2019). They are typically based on pre-trained transformers (Vaswani, et al., 2017), and are further fine-tuned with task-specific data. However, dense retrieval models trained on general datasets cannot generalize well to domain-specific IR tasks (Thakur, et al., 2021). Nevertheless, domain-specific datasets are limited in scale and diversity, restricting the creation of generalizable models (Roberts, et al., 2014; Roberts, et al., 2017; Tsatsaronis, et al., 2015; Voorhees, et al., 2021). As a result, there is a pressing need for pre-trained models that can perform well across various biomedical IR tasks.

**Figure 1.**
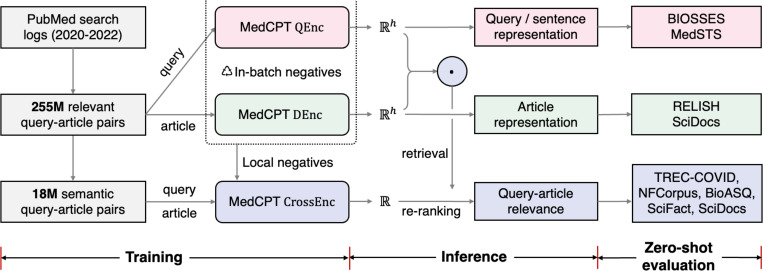
A high-level overview of this work. MedCPT contains a query encoder (QEnc), a document encoder (DEnc), and a cross-encoder (CrossEnc). The query encoder and document encoder compose of the MedCPT retriever, which is contrastively trained by 255M query-article pairs and in-batch negatives from PubMed logs. The cross-encoder is the MedCPT re-ranker, and is contrastively trained by 18M non-keyword query-article pairs and local negatives retrieved from the MedCPT retriever. MedCPT achieves state-of-the-art performance on various biomedical information retrieval tasks under zero-shot settings, including query-article retrieval, sentence representation, and article representation.

In response, we propose bioMedical Contrastive Pre-trained Transformers (MedCPT), a novel model trained with an unprecedented scale of 255M query-article pairs from PubMed search logs. MedCPT is the first biomedical IR model that includes a pair of retriever and re-ranker closely integrated by contrastive learning. Unlike previous separately developed models that have a discrepancy between the two modules (Gao, et al., 2021), MedCPT re-ranker is trained with the negative distribution sampled from the pre-trained MedCPT retriever. This matches the inference time article distribution where the MedCPT re-ranker is used to re-rank the articles returned by the MedCPT retriever. As shown in Figure 1, we perform zero-shot evaluation on a wide range of biomedical IR tasks. For document retrieval, MedCPT (330M) achieves state-of-the-art (SOTA) document retrieval performance on three individual biomedical tasks and the overall average in BEIR (Thakur, et al., 2021), outperforming much larger models such as Google’s GTR-XXL (4.8B) (Ni, et al., 2021) and OpenAI’s cpt-text-XL (175B) (Hirschman, et al., 2012). For article representation, we also show that the MedCPT article encoder sets new SOTA performance on the RELISH similar article dataset (Brown, et al., 2019) and the MeSH prediction task in SciDocs (Cohan, et al., 2020). For sentence representation, MedCPT performs the best or second best among compared methods on the BIOSESS (Sogancioglu, et al., 2017) and MedSTS (Wang, et al., 2020) for semantic evaluations. As such, MedCPT can be readily applied to a variety of biomedical applications such as searching relevant documents, retrieving similar sentences, recommending related articles, as well as providing domain-specific retrieval-augmentation for large language models (Jin, et al., 2023).

## Materials and Methods

### Query-article relevance data collection from PubMed search logs

We collected anonymous query-article clicks in PubMed search logs in three years (2020–2022) to train MedCPT. The raw logs contain 167M unique queries and 23M unique PubMed articles. We first filtered the navigational queries like author and journal title searches with Field Sensor (Yeganova, et al., 2018). After filtering, there are 87M informational queries and 17M articles. Based on the user click information, we generated 255M relevant query-article pairs to train the MedCPT retriever. However, most of such queries are short keywords, and matching them to the clicked articles is a relatively simple task. As such, we use a difficult subset that requires better semantic understanding to train the MedCPT re-ranker, which is aimed to distinguish harder negatives among the top-ranking articles returned by the retriever. Specifically, we further filtered out 79M keyword queries from the informational query set, which are defined as either having only one word or all of the clicked articles containing exact mentions of the whole input query. In the end, there are 7.7M non-keyword (e.g., short sentences) queries and 5.2M articles, from which we generated 18.3M relevant query-article pairs to train the MedCPT reranker.

### MedCPT architecture

MedCPT includes a first-stage retriever and a second-stage re-ranker. The retriever includes a query encoder (QEnc in Figure 1) and a document encoder (DEnc). This bi-encoder architecture is scalable because millions of articles can be encoded offline, and only one encoding computation for the query and a nearest neighbor search are required during real-time inference. The re-ranker is a cross-encoder (CrossEnc) that is computationally more expensive but also more accurate due to the cross-attention computation between query and article tokens. It will only be applied on the top articles returned by the retriever and generate the final article ranking.

### MedCPT retriever

The MedCPT retriever contains QEnc and DEnc, both of which are Transformer (Trm) encoders initialized by PubMedBERT (Gu, et al., 2021). It represents the query q and document d by:

$$\;E(q)=\text{QEnc}(q)=\text{Trm}{\left([\text{CLS}]q[\text{SEP}]\right)}_{\left[\text{CLS}\right]}\in {\mathbb{R}}^{h}\;E(d)=\text{DEnc}(d)=\text{Trm}{\left(\left[\text{CLS}]d^{\text{title}}[\text{SEP}]d^{\text{abstract}}[\text{SEP}\right]\right)}_{\left[\text{CLS}\right]}\in {\mathbb{R}}^{h}$$

where [CLS] and [SEP] are the special tokens used in BERT. h is the hidden. $d^{\text{title}}$ and $d^{\text{abstract}}$ denote the title and abstract. Then, the relevance is calculated as: $Rel(q,d)=E{\left(q\right)}^{T}E(d)\in \mathbb{R}$.

**Figure 2.**
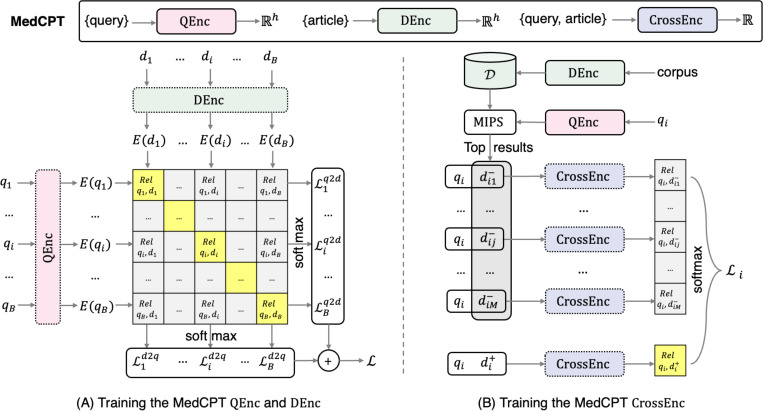
Overview of the MedCPT training process. (A) Training the MedCPT query encoder (QEnc) and document encoder (DEnc) using a contrastive loss with query-document pairs and in-batch negatives; (B) Training the MedCPT cross-encoder (CrossEnc) using a contrastive loss with non-keyword query-article pairs and local negatives derived from the MedCPT retriever. Models in dashed and solid lines denote un-trained and pre-trained, respectively. MIPS: maximum inner product search.

As shown in Figure 2 (A), to train the MedCPT retriever, each instance has a query q, a clicked document d, and the number of clicks c. Each mini-batch contains |B| instances, denoted as ${\left[q_{i},d_{i},c_{i}\right]}_{i=1}^{\left|B\right|}$. We use contrastive loss with in-batch negatives (Karpukhin, et al., 2020; Neelakantan, et al., 2022). Specifically, we first generate all $E\left(q_{i}\right)$ and $E\left(d_{i}\right)$ from QEnc and DEnc, where $d_{i}$ is a relevant document for $q_{i}$. We assume that the (|B|−1) other documents $\left[d_{j}|j\neq i\right]$ in the mini-batch are irrelevant documents for $q_{i}$. Similarly, we also consider the (|B|−1) other queries $\left[q_{j}|j\neq i\right]$ in the mini-batch as irrelevant queries for $d_{i}$. For the training instance i, we calculate its query-to-document loss $L_{i}^{q2d}$ and document-to-query loss $L_{i}^{d2q}$ by:

$$L_{i}^{q2d}=-\log\left(\frac{\exp\left(E{\left(q_{i}\right)}^{T}E\left(d_{i}\right)\right)}{\sum_{m=1}^{\left|B\right|}\exp\left(E{\left(q_{i}\right)}^{T}E\left(d_{m}\right)\right)}\right)\;\text{and}\;L_{i}^{d2q}=-\log\left(\frac{\exp\left(E{\left(q_{i}\right)}^{T}E\left(d_{i}\right)\right)}{\sum_{m=1}^{\left|B\right|}\exp\left(E{\left(q_{m}\right)}^{T}E\left(d_{i}\right)\right)}\right)$$

We further weight the loss of instances by their clicks: $L_{B}^{q2d}=\sum_{i=1}^{\left|B\right|}w_{i}L_{i}^{q2d}$ and $L_{B}^{d2q}=\sum_{i=1}^{\left|B\right|}w_{i}L_{i}^{d2q}$, where $w_{i}=\frac{\log_{2}\left(c_{i}+1\right)}{\sum_{k=1}^{\left|B\right|}\log_{2}\left(c_{k}+1\right)}$. The final loss $L_{B}$ of the mini-batch is their weighted sum and is optimized by gradient-based methods.

### MedCPT re-ranker

The MedCPT re-ranker is a cross-encoder, denoted as CrossEnc. Similarly, CrossEnc is also initialized with PubMedBERT. The MedCPT re-ranker predicts the relevance between a query q and a document d by passing them into a single CrossEnc.

Specifically,

$$Rel(q,d)=\text{CrossEnc}(q,d)=W^{T}\;\text{Trm}{\left([\text{CLS}]\;q\;[\text{SEP}]\;d\;[\text{SEP}]\right)}_{\left[\text{CLS}\right]}+b\in \mathbb{R}$$

where $W\in {\mathbb{R}}^{h}$ and b∈ℝ are trainable parameters.

As shown in Figure 2 (B), for training the MedCPT re-ranker, each instance has a query $q_{i}$, a clicked document $d_{i}^{+}$, and a list of M irrelevant (not clicked) documents $\left\{d_{ij}^{-}|j=\right.1,2,3,\ldots ,M\}$. Following (Gao, et al., 2021), we use local negatives to train the MedCPT re-ranker instead of in-batch negatives. Specifically, unlike the in-batch negative documents used by the MedCPT retriever that are approximately random samples, the local negative documents are sampled from rank e to rank f in the top retrieved documents by the pre-trained MedCPT retriever through a maximum inner product search, which ensures that the MedCPT re-ranker can distinguish the hard negatives returned by the retriever. The loss $L_{i}$ for the instance is a negative log-likelihood loss:

$$L_{i}=-\log\left(\frac{\exp\left(\text{CrossEnc}\left(q_{i},d_{i}^{+}\right)\right)}{\exp\left(\text{CrossEnc}\left(q_{i},d_{i}^{+}\right)\right)+\sum_{j=1}^{M}\exp\left(\text{CrossEnc}\left(q_{i},d_{ij}^{-}\right)\right)}\right)$$

We take a weighted sum of the instance-level loss and optimize the final loss by gradient-based methods. More details on MedCPT inference and configuration are shown in Appendix A.

## Results

### MedCPT achieves state-of-the-art performance on biomedical IR tasks

Benchmarking-IR (BEIR) (Thakur, et al., 2021) is a standardized evaluation benchmark for zero-shot IR systems. We evaluate MedCPT with all five biomedical tasks in the BEIR benchmark. Appendix C describes the evaluation details and Table 1 shows the evaluation results.

First, MedCPT improves its initialization PubMedBERT by huge margins, where the latter basically fails on the retrieval tasks. Overall, MedCPT sets new SOTA performance on 3/5 tasks, surpassing compared sparse (Dai and Callan, 2020; Nogueira, et al., 2019; Zhang, et al., 2015), dense (Hofstätter, et al., 2021; Izacard, et al., 2021; Karpukhin, et al., 2020; Xiong, et al., 2020), and late-interaction (Khattab and Zaharia, 2020) retrievers on all of the compared tasks. As shown in the BEIR paper, BM25 is a strong baseline that is generalizable to biomedical IR tasks. Notably, MedCPT is still better than BM25 with cross-encoder in 4/5 of the evaluated tasks, showing its effectiveness at retrieving relevant articles for biomedical queries. BM25 with re-ranker is only better on the TREC-COVID dataset, which might be due to annotation biases (Thakur, et al., 2021). We further compare MedCPT with more recent large dual retriever models, represented by Google’s GTR and OpenAI’s cpt-text, both of which have model sizes ranging from millions to billions of parameters. MedCPT is able to outperform all sizes of the GTR model. While the GPT-3 (Brown, et al., 2020) sized (175B) cpt-text-XL is better than MedCPT on NFCorpus, MedCPT outperforms cpt-text-XL on TREC-COVID and SciFact despite being about 500 times smaller. This indicates that small models trained on domain-specific datasets can still have better in-domain zero-shot performance than much larger general domain retrievers.

### MedCPT generates better biomedical article representations

We evaluate the MedCPT article encoder on the RELISH article similarity task (Brown, et al., 2019). RELISH is an expert-annotated dataset that contains 196k article-article relevance annotations for 3.2k query articles, as described in Appendix D. Table 2 shows the evaluation results on RELISH. The MedCPT article encoder (DEnc) outperforms all other models, including SPECTER (Cohan, et al., 2020) and SciNCL (Ostendorff, et al., 2022) that are specifically trained with article-article citation information. Compared to its base PubMedBERT model, the MedCPT article encoder improves by over 10% performance. We also evaluate the MedCPT article encoder on SciDocs (Cohan, et al., 2020) as described in Appendix E, which contains all scientific domains from biomedicine to engineering. The MedCPT article encoder achieves SOTA performance on the MeSH prediction subtask and is comparable to SOTA methods on the overall score, showing its effectiveness on biomedical tasks and generalizability to other scientific domains.

### MedCPT generates better biomedical sentence representations

We evaluate the MedCPT query encoder on two datasets for sentence similarities: BIOSSES in the biomedical domain (Sogancioglu, et al., 2017) and MedSTS in the clinical domain (Wang, et al., 2020). Appendix F introduces the evaluation details and Table 3 shows the evaluation results. On BIOSSES, MedCPT performs the best among all compared models, surpassing the second SciNCL by 5% relative performance (0.893 vs. 0.847). On the MedSTS dataset, MedCPT ranks the second and the performance is comparable to the highest-ranking model BioSentVec (Chen, et al., 2019) (0.765 vs. 0.767), which uses an external clinical corpus MIMIC-III (Johnson, et al., 2016) for its model training. Overall, our results show that the MedCPT query encoder can effectively encode biomedical and clinical sentences that reflect their semantic similarities.

## Discussions

MedCPT is only trained with query-article click data derived from PubMed user logs, but it generalizes well and achieves the SOTA performance on many biomedical IR tasks in the BEIR benchmark, which indicates that query-article pairs in the PubMed search logs can serve as high-quality training data for serving general-purpose information needs in biomedicine. Furthermore, while not being explicitly trained with query similarity and article similarity data, the MedCPT query encoder and article encoder still achieve the SOTA performance on sentence similarity and article similarity tasks, respectively. This shows that the contrastive objective can train not only a dense retriever, but can also train the individual query and document encoders to perform tasks related to information-seeking behaviors. As such, MedCPT has broad implications in a variety of real-world scenarios: enhancing algorithms for biomedical literature search such as PubMed’s Best Match (Fiorini, et al., 2018), where case studies in Appendix G show that MedCPT retrieves more semantically relevant articles than other commonly used literature search engines; improving similar article recommendation algorithms in literature search (Lin and Wilbur, 2007); facilitating sentence-to-sentence retrieval tasks such as sentence-level literature search (Allot, et al., 2019).

Although transformer-based retrieval and re-ranking models such as MedCPT can return more comprehensive results, they are not as controllable or explainable as sparse retrievers such as BM25. For example, when user searches the gene “MAP3K3”, MedCPT will also return articles that only contain “MAP3K7”, which might not be the original information need. In addition, the semantic similarity scores between a query article pair are not explainable. As such, one potential future direction is to develop hybrid dense-sparse retrieval systems that can harvest the advantages from both approaches (Ma, et al., 2020; Shin, et al., 2023).

To summarize, we use large-scale PubMed logs to contrastively train MedCPT, the first integral retriever-reranker model for biomedical information retrieval. Systematic zero-shot evaluations show that MedCPT achieves the highest performance for six different biomedical information retrieval tasks, including query-to-article retrieval, semantic article and sentence representation. We anticipate that MedCPT will have a broad range of applications and significantly enhance access to biomedical information, making it a valuable tool for researchers and practitioners alike.

## Supplementary Material

Supplement 1

## Figures and Tables

**Table 1.** Zero-shot performance of MedCPT on biomedical subtasks of the BEIR benchmark. **Bolded numbers**, underlined, and *italicized* numbers denote the **highest**, 2^nd^ highest, and *3*^*rd*^ *highest*, respectively. COVID: TREC-COVID; NFC: NFCorpus; Avg.: average.

Method	Size	COVID	NFC	BioASQ	SciFact	SciDocs	Avg.
**Sparse retrievers**							
BM25	-	0.656	0.325	0.465	0.665	0.158	*0.454*
BM25 + MiniLM	66M	**0.757**	0.350	0.523	0.688	*0.166*	0.497
DeepCT	110M	0.406	0.283	0.407	0.630	0.124	0.370
SPARTA	110M	0.538	0.301	0.351	0.582	0.126	0.380
docT5query	220M	0.713	0.328	0.431	0.675	0.162	0.462
**Dense retrievers**							
DPR	110M	0.332	0.189	0.127	0.318	0.077	0.209
ANCE	110M	0.654	0.237	0.306	0.507	0.122	0.365
TAS-B	66M	0.481	0.319	0.383	0.643	0.149	0.395
GenQ	220M	0.619	0.319	0.398	0.644	0.143	0.425
Contriever	110M	0.596	0.328	-	0.677	0.165	-
Contriever + MiniLM	176M	*0.701*	0.344	-	0.692	0.171	-
ColBERT	110M	0.677	0.305	*0.474*	0.671	0.145	*0.454*
**Large language model retrievers**							
Google GTR-Base	110M	0.539	0.308	0.271	0.600	0.149	0.373
Google GTR-Large	335M	0.557	0.329	0.320	0.639	0.158	0.401
Google GTR-XL	1.24B	0.584	0.343	0.317	0.635	0.159	0.408
Google GTR-XXL	*4.80B*	0.501	0.342	0.324	0.662	0.161	0.398
OpenAI cpt-text-S	300M	0.679	0.332	-	0.672	-	-
OpenAI cpt-text-M	1.20B	0.585	*0.367*	-	0.704	-	-
OpenAI cpt-text-L	6.00B	0.562	0.380	-	*0.744*	-	-
OpenAI cpt-text-XL	**175B**	0.649	**0.407**	-	0.754	-	-
**MedCPT**							
MedCPT	330M	0.709	0.355	**0.553**	**0.761**	**0.172**	**0.510**
MedCPT (retriever only)	220M	0.697	0.340	0.332	0.724	0.123	0.443
MedCPT w/o contrastive pre-training (PubMedBERT)	110M	0.059	0.015	-	0.010	0.004	-

**Table 2.** Evaluation results of the MedCPT article encoder on the RELISH dataset. **Bolded numbers**, underlined, and *italicized* numbers denote the **highest**, 2nd highest, and *3rd highest*, respectively. All numbers are percentages. Avg.: average.

Method	MAP			NDCG			Avg.
	@5	@10	@15	@5	@10	@15	
Random	79.33	77.22	75.41	80.70	77.67	76.40	77.79
**Sparse retrievers**							
BM25	88.91	86.72	84.54	89.48	87.39	86.21	87.21
PMRA	90.30	87.57	85.75	90.95	88.40	87.45	88.40
**Non-BERT embedding-based models**							
fastText	85.75	82.81	81.79	86.79	83.79	83.12	84.01
BioWordVec	89.84	86.51	84.67	89.90	86.67	85.53	87.19
InferSent	85.21	82.16	80.41	86.56	83.31	82.35	83.33
WikiSentVec	87.92	85.23	83.40	88.65	85.74	84.81	85.96
BioSentVec	90.76	88.10	86.16	90.05	87.76	86.89	88.29
LDA	85.44	82.66	80.36	86.51	82.91	81.31	83.20
Doc2Vec	86.23	84.74	83.39	86.55	84.70	84.09	84.95
**BERT-based models**							
BioBERT	88.14	85.81	83.90	88.97	86.29	85.10	86.37
PubMedBERT	83.69	81.07	79.53	85.47	82.39	81.41	82.26
SPECTER	*92.27*	*90.00*	*88.36*	*91.47*	*89.12*	*88.42*	*89.94*
SciNCL	94.72	92.74	91.14	93.67	91.91	90.94	92.52
MedCPT DEnc	**95.58**	**93.99**	**92.39**	**94.78**	**93.12**	**92.43**	**93.72**

**Table 3.** Evaluation results (Pearson’s correlation coefficients) of the MedCPT query encoder on the BIOSSES and MedSTS datasets. **Bolded numbers**, underlined, and *italicized* numbers denote the **highest**, 2nd highest, and *3rd highest*, respectively. All numbers are percentages.

Model	BIOSSES	MedSTS
**Non-BERT embedding-based models**		
BioWordVec	0.694	0.747
USE	0.345	0.714
BioSentVec (PubMed)	*0.817*	0.750
BioSentVec (MIMIC-III)	0.350	*0.759*
BioSentVec (PubMed + MIMIC-III)	0.795	**0.767**
**BERT-based models**		
PubMedBERT	0.528	0.521
Clinical BERT	0.556	0.525
SPECTER	0.694	0.702
SciNCL	0.847	0.706
MedCPT QEnc	**0.893**	0.765
