# Supplementary material for: MedCPT: Contrastive Pre-trained Transformers with Large-scale PubMed Search Logs for Zero-shot Biomedical Information Retrieval
Source: ArXiv. 2023 Oct 4:arXiv:2307.00589v2. Preprint. [Version 2] (PMC12478430)
Supplement: Supplement 1 [file NIHPP2307.00589v2-supplement-1.pdf]

Appendix A: MedCPT Inference

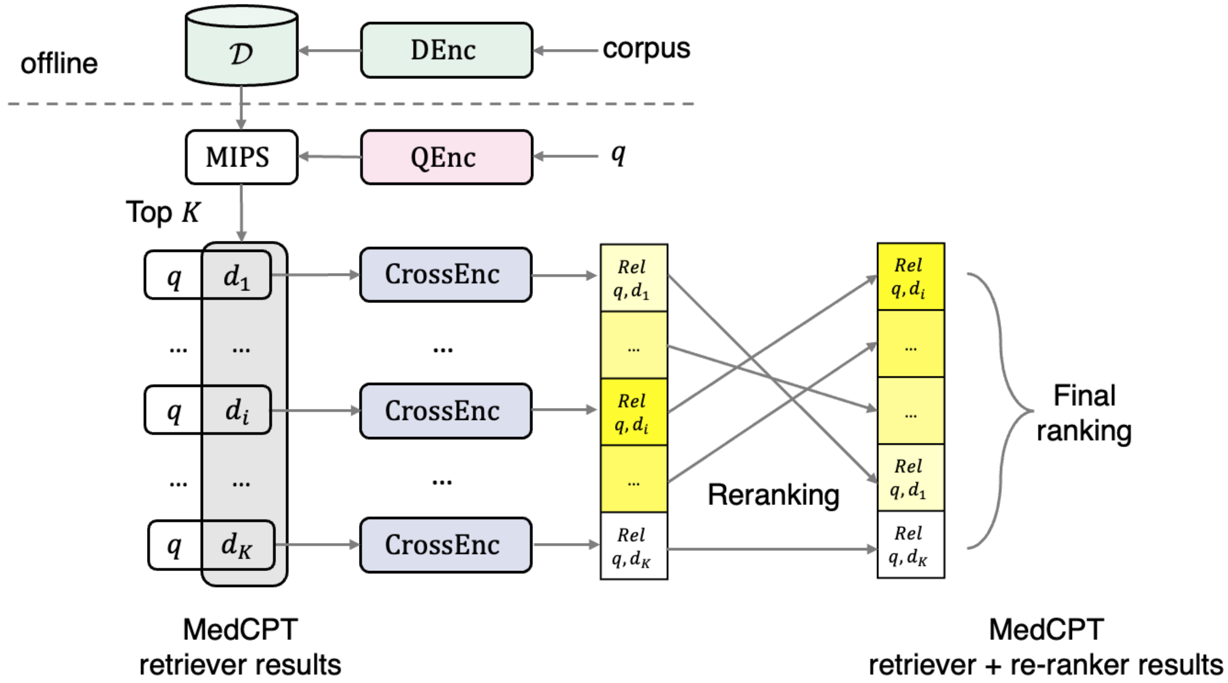

Figure S1. Architecture for inference with pre-trained MedCPT. A test query q is fed to the MedCPT query encoder, and its representation will be matched against the corpus representations using MIPS. The corpus representations are calculated and saved off-line. Top K retrieved articles will be re-ranked by the MedCPT cross-encoder. MIPS: maximum inner product search.

As shown in Figure S1, when applying the pre-trained MedCPT to downstream tasks in a zero-short fashion, we need to first encode the task corpus. Specifically, we use DEnc to process each article d_i in the task corpus, getting their representations $E(d_i) \in \mathbb{R}^h$. We save the representations of the entire corpus, denoted as:

$$\mathcal{D} = [E(d_1), E(d_2), \dots, E(d_N)] \in \mathbb{R}^{N \times h}$$

where N is the size of articles in the corpus. This step only needs to be done once in the offline setting.

Then, each input query q is fed into QEnc, which generates its representative $E(q)$. We conduct a MIPS between $E(q)$ and $\mathcal{D}$ to find the top-Kmost similar articles to the input query:

$$d_1^q, d_2^q, \dots, d_K^q = \mathrm{MIPS}(E(q), \mathcal{D})$$

We apply $\mathrm{CrossEnc}$ to score the relevance between q and each relevant article candidates in $d_1^q, d_2^q, \dots, d_K^q$ retrieved from the previous step:

$$Rel(q, d_i^q) = \mathrm{CrossEnc}(q, d_i^q)$$

Finally, we sort the retrieved articles by $Rel(q, d_i^q)$ from the highest to the lowest and return the sorted results.

We implemented the MedCPT using PyTorch (Paszke, et al., 2019) and the Hugging Face transformers library (Wolf, et al., 2020). The hidden dimension for MedCPT $h = 768$ as in the BERT-base configuration. We use the Adam optimizer (Kingma and Ba, 2014) without weight decay to train both the retriever and the re-ranker, where we set the learning rate 2e-5 and epsilon 1e-8. For the MedCPT retriever, $B = 32$ and $\alpha = 0.8$, and we also apply gradient accumulation of 8 steps. We train the retriever for 100k steps with 10k warm-up steps. For the MedCPT re-ranker: $M = 31$, $e = 50$, $f = 200$. We train the re-ranker for 10k steps with 1,000 warm-up steps. We apply cosine learning rate schedule after the warm-up steps. During inference, N and K vary for specific tasks. We implemented MIPS with the FlatIP index of the Faiss library (Johnson, et al., 2019).

Appendix B: Compared methods

Sparse retrievers

Sparse retrievers, as known as lexical retrievers, match the queries and documents with overlapped terms. BM25 (Robertson and Zaragoza, 2009), a widely used lexical retriever, represents queries and documents as bag-of-words, and scores the relevance based on term frequency and inverse document frequency. DeepCT (Dai and Callan, 2020) uses contextualized term weights predicted by BERT. SPARTA (Zhang, et al., 2015) pre-computes contextualized matching weights for each possible term and the document, resulting in a sparse vector that has the size dimension as the BERT vocabulary. DocT5query (Nogueira, et al., 2019) performs document expansion with T5-generated (Raffel, et al., 2020) queries for the document. The PubMed Related Articles algorithm (Lin and Wilbur, 2007) uses a probabilistic topic-based model to compute the content similarity between two given articles.

Dense retrievers

Dense retrievers first encode both the queries and documents into low-dimensional dense vectors, and then perform nearest neighbor search to find the relevant documents for a given query. Depending on the encoder architecture, we broadly classify them into non-BERT embedding models and BERT-based dense retrievers.

Non-BERT embedding models include BioWordVec (Zhang, et al., 2019) which is a biomedical version of word2vec (Mikolov, et al., 2013), FastText (Bojanowski, et al., 2017), a linear model on the N-gram features of the input texts, Sent2vec (Pagliardini, et al., 2018) and its biomedical version BioSentVec (Chen, et al., 2019), the LDA topic model (Blei, et al., 2003), doc2vec (Le and Mikolov, 2014), InferSent (Conneau, et al., 2017), and Universal Sentence Encoder (USE) (Cer, et al., 2018). BERT-based dense retrievers: DPR (Karpukhin, et al., 2020) is a bi-encoder retriever trained by in-batch negatives and BM25 hard negatives. ANCE (Xiong, et al., 2020) improves the DPR training by using hard negatives from an approximate nearest neighbor index of the corpus. TAS-B (Hofstätter, et al., 2021) is a bi-encoder retriever distilled from a cross-encoder and ColBERT (Khattab and Zaharia, 2020) with balanced topic aware sampling. GenQ (Thakur, et al., 2021) is domain-adaptation method that trains a dense retriever with synthetic query-document pairs generated by T5 (Raffel, et al., 2020). Contriever (Izacard, et al., 2021) is a contrastively pre-trained dense retriever in the general domain with carefully engineered positive and negative query-document pairs. ColBERT (Khattab and Zaharia, 2020) is a late-interaction retriever that computes and matches the contextualized representations of each token in the query and document. In addition, we compare MedCPT with several off-the-shelf BERT models that are biomedical domain-

specific, including its base model PubMedBERT (Gu, et al., 2021), BioBERT (Lee, et al., 2020), SPECTER (Cohan, et al., 2020), and SciNCL (Ostendorff, et al., 2022).

Large language model retrievers

We also compare MedCPT with two large language model retrievers: Google's GTR (Ni, et al., 2021) and OpenAI's cpt-text (Hirschman, et al., 2012). Unlike most dense retrievers that are based on the BERT-base model of 110M parameters, GTR and cpt-text use much larger language model encoders. Specifically, GTR is based on T5 (Raffel, et al., 2020) and its largest variant has 4.8B parameters, while cpt-text is based on GPT-3 (Brown, et al., 2020) and its largest variant has 175B parameter. Both GTR and cpt-text are pre-trained by large-scale Web corpora with in-batch negatives, and are further fine-tuned with supervised datasets such as MS MARCO (Bajaj, et al., 2016). In comparison, MedCPT is only trained by the user click data from PubMed logs without using any supervised datasets.

Appendix C: Evaluation details on BEIR

We evaluate MedCPT on five biomedical tasks in the BEIR benchmark: TREC-COVID (Voorhees, et al., 2021), NFCorpus (Boteva, et al., 2016), BioASQ (Tsatsaronis, et al., 2015), SciFact (Wadden, et al., 2020), and SciDocs (Cohan, et al., 2020). TREC-COVID (Voorhees, et al., 2021) contains questions about the COVID-19 pandemic and uses the CORD-19 corpus (Wang, et al., 2020) as the document collection for retrieval. NFCorpus (Boteva, et al., 2016) collects natural language queries and relevant articles from the NutritionFacts.org site. BioASQ (Tsatsaronis, et al., 2015) is a community challenge for biomedical question answering, where the task used in BEIR is to retrieve relevant articles from PubMed for a given question. SciFact (Wadden, et al., 2020) is a scientific claim verification dataset, which contains a retrieval subtask and a veracity prediction subtask. BEIR uses the retrieval subtask of SciFact, where the objective is to find relevant articles that can be used to verify a given claim. SciDocs (Cohan, et al., 2020) is a benchmark for evaluating scientific article representation models. BEIR uses its citation prediction subtask, where the goal is to retrieve relevant citations for a given article. We use the official evaluation library for BEIR and report the normalized discounted cumulative gain at rank 10 (NDCG@10).

We compared it with various baselines, including its initialization model PubMedBERT (Gu, et al., 2021), BM25 (Robertson and Zaragoza, 2009) and BM25 with the MiniLM re-ranker (Wang, et al., 2020), sparse retrievers such as DeepCT (Dai and Callan, 2020), SPARTA (Zhang, et al., 2015), and docT5query (Nogueira, et al., 2019), dense retrievers such as DPR (Karpukhin, et al., 2020), ANCE (Xiong, et al., 2020), TAS-B (Hofstätter, et al., 2021), GenQ (Thakur, et al., 2021), Contriever (Izacard, et al., 2021), large language model generated embeddings such as Generalizable T5-based dense Retrievers (GTR) (Ni, et al., 2021) and cpt-text (Neelakantan, et al., 2022). Details of the compared methods are described in Appendix B.

Appendix D: Evaluation details on RELISH

Following the dataset split and evaluation settings of (Zhang, et al., 2022), we use article embeddings generated by the MedCPT article encoder to calculate the article-pair similarity and evaluate ranking quality by mean average precision (MAP) and NDCG at 5, 10, and 15.

For comparison, we also list the model performance reported in (Zhang, et al., 2022), including a random baseline, term-based retrievers such as BM25 (Robertson and Zaragoza, 2009) and PubMed Related Articles (PMRA) (Lin and Wilbur, 2007), embedding-based retrievers such as fastText (Bojanowski, et al., 2017), BioWordVec (Zhang, et al., 2019), InferSent (Conneau, et al., 2017), Sent2vec (Pagliardini, et al., 2018), BioSentVec (Chen, et al., 2019), document embedding models such as LDA (Blei, et al., 2003) and doc2vec (Le and Mikolov, 2014), and BERT-based retrievers such as BioBERT (Lee, et al., 2020), PubMedBERT (Gu, et al., 2021), SPECTER (Cohan, et al., 2020), and SciNCL (Ostendorff, et al., 2022). Details of the compared methods are described in Appendix B.

Appendix E: Evaluation details on SciDocs

SciDocs is an evaluation framework for measuring the effectiveness of scientific paper embeddings. It includes several subtasks, such as classification of article topics, predicting user activity and citation, and also article recommendation. On SciDocs, we compare the MedCPT article encoder with various text representation models, including a random baseline, doc2vec (Le and Mikolov, 2014), FastText (Bojanowski, et al., 2017), SIF (Arora, et al., 2017), ELMo (Peters, et al., 2018), Citeomatic (Bhagavatula, et al., 2018), SGC (Wu, et al., 2019), and BERT-based models such as SciBERT (Beltagy, et al., 2019), Sent-BERT (Reimers and Gurevych, 2019), PubMedBERT (Gu, et al., 2021), SPECTER (Cohan, et al., 2020), SciNCL (Ostendorff, et al., 2022). We use the official evaluation library for SciDocs[1] and report the returned metrics for each subtask. Details of the compared methods are described in Appendix B.

Table S1 shows the evaluation results on the SciDocs benchmark. For the average performance on subtasks, the MedCPT article encoder is better than all other compared baselines except SPECTER and SciNCL. This is not surprising since (1) SciDocs includes articles in other scientific disciplines than biomedicine and (2) SPECTER and SciNCL are trained with article-article citation information and optimized for the SciDocs benchmark. However, MedCPT is still able to surpass SPECTER and SciNCL on the MeSH classification sub-task, which only contains biomedical articles. Overall, the MedCPT article encoder is comparable to SPECTER and SciNCL for scientific article representation, and better for biomedical article representation.

[1] https://github.com/allenai/scidocs

SciDocs Task	Classification		User activity prediction				Citation prediction				Recomm.		Avg.
	MAG	MSH	Co-View		Co-Read		Cite		Co-Cite				
	F1	F1	M.	N.	M.	N.	M.	N.	M.	N.	N.	P@1	
Random	4.8	9.4	25.2	51.6	25.6	51.9	25.1	51.5	24.9	51.4	51.3	16.8	32.5
Doc2vec	66.2	69.2	67.8	82.9	64.9	81.6	65.3	82.2	67.1	83.4	51.7	16.9	66.6
Fasttext-sum	78.1	84.1	76.5	87.9	75.3	87.4	74.6	88.1	77.8	89.6	52.5	18.0	74.1
SIF	78.4	81.4	79.4	89.4	78.2	88.9	79.4	90.5	80.8	90.9	53.4	19.5	75.9
ELMo	77.0	75.7	70.3	84.3	67.4	82.6	65.8	82.6	68.5	83.8	52.5	18.2	69.0
Citeomatic	67.1	75.7	81.1	90.2	80.5	90.2	86.3	94.1	84.4	92.8	52.5	17.3	76.0
SGC	76.8	82.7	77.2	88.0	75.7	87.5	91.6	96.2	84.1	92.5	52.7	18.2	76.9
SciBERT	79.7	80.7	50.7	73.1	47.7	71.1	48.3	71.7	49.7	72.6	52.1	17.9	59.6
Sent-BERT	80.5	69.1	68.2	83.3	64.8	81.3	63.5	81.6	66.4	82.8	51.6	17.1	67.5
SPECTER	**82.0**	*86.4*	83.6	91.5	84.5	92.4	*88.3*	*94.9*	88.1	94.8	**53.9**	**20.0**	80.0
SciNCL	81.4	88.7	**85.3**	**92.3**	**87.5**	**93.9**	**93.6**	**97.3**	**91.6**	**96.4**	**53.9**	*19.3*	**81.8**
PubMedBERT	77.3	80.5	47.4	70.2	45.2	68.3	40.6	65.4	44.8	68.4	51.8	17.4	56.4
MedCPT DEnc	*80.3*	**89.9**	*82.3*	*90.8*	*83.1*	*91.6*	83.2	92.5	*85.1*	*93.5*	*52.9*	18.5	*78.6*

Table S1. Evaluation results of the MedCPT article encoder on the SciDocs benchmark. MeSH classification is the only biomedical task. **Bolded numbers**, underlined, and *italicized* numbers denote the **highest**, 2nd highest, and *3rd highest*, respectively. All numbers are percentages. M: MAP; N: NDCG. Recomm.: recommendation. Avg.: average.

Appendix F: Evaluation details on BIOSSES and MedSTS

The evaluation is conducted under the unsupervised (zero-shot) setting, where we directly apply the MedCPT query encoder model to test set instances without any model retraining or fine-tuning. We follow the evaluation settings in (Chen, et al., 2019) and report Pearson's correlation coefficients between the model predictions and the ground truth scores. For comparison, we include SOTA methods such as BioWordVec (Chiu, et al., 2016), Universal Sentence Encoder (USE) (Cer, et al., 2018), BioSentVec trained with different corpora (Chen, et al., 2019), and BERT-based models such as PubMedBERT (Gu, et al., 2021), Clinical BERT (Alsentzer, et al., 2019), SPECTER (Cohan, et al., 2020),

SciNCL (Ostendorff, et al., 2022). Details of the compared methods are described in Appendix B.

Appendix G: Case studies

We conduct three case studies by comparing MedCPT results with widely used web-based literature search tools, including PubMed (with Best Match ranking (Fiorini, et al., 2018)), Google Scholar[2], and Semantic Scholar[3]. Unlike the standardized biomedical IR tasks where the queries are mostly natural language sentences or questions, we choose to evaluate using short phrases (keyword combinations) that require semantic understanding in this section. We don't test on full sentences because it would be unfair for web-based search engines that are not optimized for such usage. Table S2 shows the top-3 results returned by different tools.

Query case 1 is "lead heart damage". In this query, the word "lead" most likely means the metal "lead" or the wire / cable that is used in an implanted device, but much less likely to denote the verb as in "lead to". However, "lead" is matched to "lead to" by PubMed, Google Scholar, and Semantic Scholar. MedCPT, on the other hand, matches all "lead" to the metal or a medical device. Although some titles don't explicitly contain "heart", the corresponding articles are about heart damage.

Query case 2 is "postpartum depression syndrome". In this query, the word "syndrome" is simply used to modify "postpartum depression" and does not denote any other "syndrome". The word can be neglected when retrieving relevant articles because it's not common usage. However, most web-based search engines map "syndrome" to other diseases such as "polycystic ovary syndrome" and "premenstrual syndrome" that are not part of the original information needs. MedCPT does not map the "syndrome" to other unrelated concepts, but actually tries to return more general titles that unify the query terms, i.e., "Postpartum psychiatric syndromes". MedCPT can also ignore "syndrome" as in the returned article "Postpartum Depression".

Query case 3 is "dermatologist in Germany". In this query, "Germany" should be interpreted together with "dermatologist" as the main topic. However, PubMed and Semantic Scholar mostly match "Germany" to the author's affiliation fields or the place of study. On the other hand, all three articles returned by MedCPT have exact continuous mentions of "German dermatologist(s)".

---

[2] https://scholar.google.com/
[3] https://www.semanticscholar.org/

Query	PubMed (Best Match)	Google Scholar	Semantic Scholar	PuedCPT (ours)
“lead heart damage”	Brain-**Heart** Interaction: Cardiac Complications After Stroke	New treatment strategies for alcohol-induced **heart damage**	Altered Hemodynamics and End-Organ **Damage** in **Heart** Failure	Anatomical mechanisms that cause **lead** and catheter **damage**
	An Overview of Chemical and Biological Materials **lead to** **Damage** and Repair of **Heart** Tissue	An overview of chemical and biological materials **lead to** **damage** and repair of **heart** tissue	Hibiscus sabdariffa Linn. (Roselle) protects against nicotine-induced **heart damage** in rats	**Myocardial** changes in **lead poisoning**
	Role of **Cardiac** Macrophages on **Cardiac** Inflammation, Fibrosis and Tissue Repair	Early life permethrin insecticide treatment **leads to** **heart damage** in adult rats	Use of Computational Fluid Dynamics to Analyze Blood Flow, Hemolysis and Sublethal **Damage** to Red Blood Cells in a Bileaflet Artificial **Heart** Valve	Transvenous defibrillator **lead damage**
“postpartum depression syndrome”	**Depression** During Pregnancy and **Postpartum**	History of premenstrual **syndrome** and development of **postpartum depression**: a systematic review and meta-analysis	Relationship of premenstrual **syndrome** with **postpartum depression** and mother-infant bonding.	**Postpartum psychiatric syndromes**
	Polycystic ovary **syndrome** and **postpartum depression**: A systematic review and meta-analysis of observational studies	Associations between premenstrual **syndrome** and **postpartum depression**: a systematic literature review	Polycystic Ovary **Syndrome** and **Postpartum Depression** Symptoms: A Population-Based Cohort Study.	**Postpartum psychiatric syndromes**
	Relationship of premenstrual	Polycystic ovary **syndrome** and	Polycystic ovary **syndrome** and	**Postpartum Depression**

	**syndrome** with **postpartum depression** and mother-infant bonding	**postpartum depression**: A systematic review and meta-analysis of observational studies	**postpartum depression**: a systematic review and meta-analysis of observational studies.	
"dermatologist in Germany"	[Wound treatment in diabetes patients and diabetic foot ulcers]	**Dermatology in Germany**	[External scientific evaluation of the first teledermatology app without direct patient contact in **Germany** (Online Dermatologist-AppDoc)].	[The **German** Society of **Dermatology** -- association of **German dermatologists** in Germany, Austria and Switzerland -- its position in Europe and in the world]
	Microbiome in healthy skin, update for **dermatologists**	To excise or not: impact of MelaFind on **German dermatologists'** decisions to biopsy atypical lesions	AI outperformed every **dermatologist** in dermoscopic melanoma diagnosis, using an optimized deep-CNN architecture with custom mini-batch logic and loss function	**German dermatologists** and their contributions to Turkish dermatology
	Practical management of acne for clinicians: An international consensus from the Global Alliance to Improve Outcomes in Acne	Epidemiology of contact **dermatitis**. The information network of departments of dermatology (IVDK) in **Germany**	[Why in Koenigsberg, why Samuel Jessner, why 1921? : History of the first university lectureship for sexology in **Germany**].	[Impressions and experiences of a **German dermatologist** in America].

Table S2. Top-three retrieval article titles of MedCPT and widely used literature search engines for three case study queries. The results of PubMed, Google Scholar, and Semantic Scholar were collected on Mar 25, 2023. **Bolded** texts denote lexical matching

while <u>**bolded and underlined**</u> texts denote wrong semantic matching. Titles in "[…]" denote articles in non-English languages.

Appendix H: Scaling properties of MedCPT

In Figure S2, we study the scaling properties of MedCPT. Specifically, we evaluate the MedCPT retriever performance measured by NDCG@10 on four biomedical tasks on the BEIR benchmark (TREC-COVID, SciDocs, SciFact, NFCorpus). As shown in the figure, the performance of MedCPT increases log-linearly as the number of training logs increase, and stabilize at the end of training with 255M query-article pairs. The model needs to be trained on at least 150M query-article pairs to stabilize and consistently outperform BM25, although it should be noted that BM25 appears to be a strong baseline since many IR datasets favor BM25 due to the exposure bias in annotation. Practically, 255M query-article pairs are the most we can get from the new PubMed, and training on them already takes about 1 month of computation on a server of 8 Nvidia V100 GPUs, roughly costing ~15,000 US dollars. In conclusion, it is necessary to train on large amounts of data, but the marginal gain might decrease because the performance-training size curve follows a logarithm law.

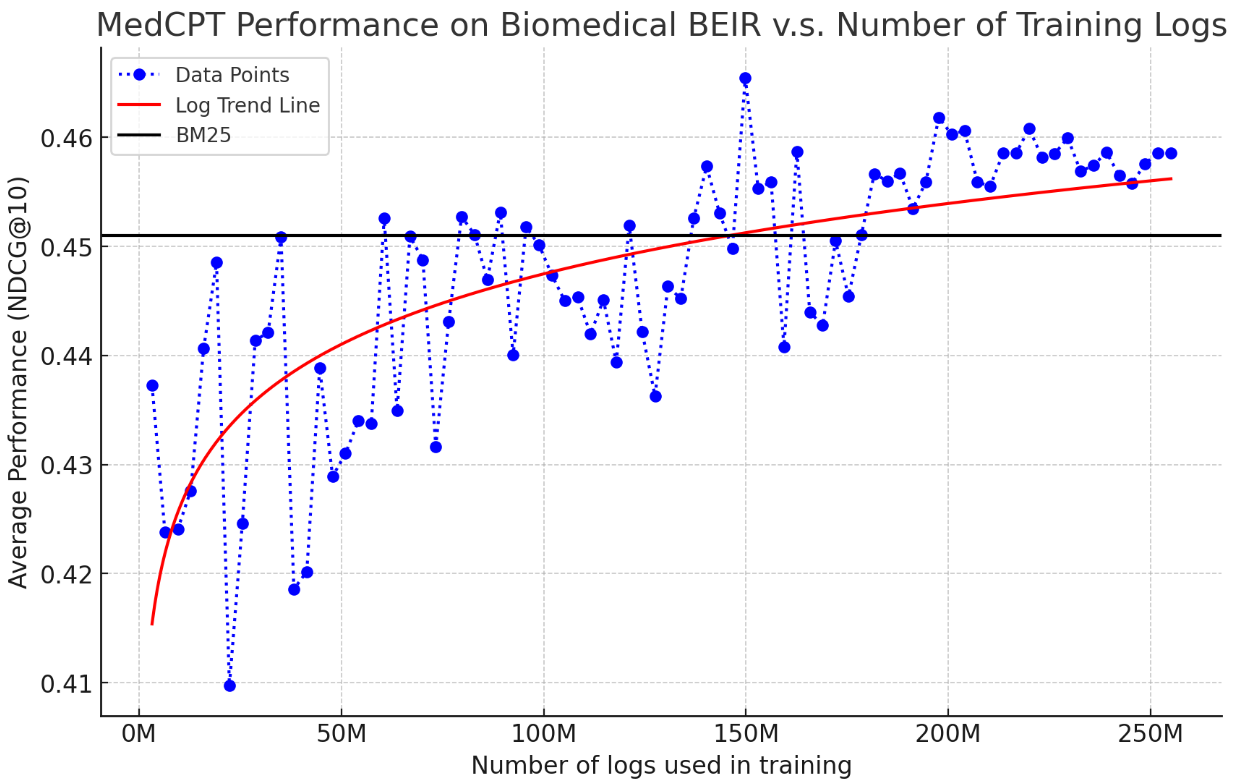

Figure S2. The average NDCG@10 performance on biomedical tasks in the BEIR benchmark of the MedCPT retrievers trained by different sizes of PubMed user logs. The performance increases log-linearly as the number of training logs increases.

References

Alsentzer, E., *et al.* Publicly Available Clinical BERT Embeddings. In, *Proceedings of the 2nd Clinical Natural Language Processing Workshop*. 2019. p. 72-78.

Arora, S., Liang, Y. and Ma, T. A simple but tough-to-beat baseline for sentence embeddings. In, *International conference on learning representations*. 2017.

Bajaj, P., *et al.* Ms marco: A human generated machine reading comprehension dataset. *arXiv preprint arXiv:1611.09268* 2016.

Beltagy, I., Lo, K. and Cohan, A. SciBERT: A Pretrained Language Model for Scientific Text. In, *Proceedings of the 2019 Conference on Empirical Methods in Natural Language Processing and the 9th International Joint Conference on Natural Language Processing (EMNLP-IJCNLP)*. 2019. p. 3615-3620.

Bhagavatula, C., *et al.* Content-Based Citation Recommendation. In, *Proceedings of the 2018 Conference of the North American Chapter of the Association for Computational Linguistics: Human Language Technologies, Volume 1 (Long Papers)*. 2018. p. 238-251.

Blei, D.M., Ng, A.Y. and Jordan, M.I. Latent dirichlet allocation. *Journal of machine Learning research* 2003;3(Jan):993-1022.

Bojanowski, P., *et al.* Enriching word vectors with subword information. *Transactions of the association for computational linguistics* 2017;5:135-146.

Boteva, V., *et al.* A full-text learning to rank dataset for medical information retrieval. In, *Advances in Information Retrieval: 38th European Conference on IR Research, ECIR 2016, Padua, Italy, March 20–23, 2016. Proceedings 38*. Springer; 2016. p. 716-722.

Brown, T., *et al.* Language models are few-shot learners. *Advances in neural information processing systems* 2020;33:1877-1901.

Cer, D., *et al.* Universal sentence encoder. *arXiv preprint arXiv:1803.11175* 2018.

Chen, Q., Peng, Y. and Lu, Z. BioSentVec: creating sentence embeddings for biomedical texts. In, *2019 IEEE International Conference on Healthcare Informatics (ICHI)*. IEEE; 2019. p. 1-5.

Chiu, B., *et al.* How to train good word embeddings for biomedical NLP. In, *Proceedings of the 15th workshop on biomedical natural language processing*. 2016. p. 166-174.

Chowdhery, A., *et al.* Palm: Scaling language modeling with pathways. *arXiv preprint arXiv:2204.02311* 2022.

Cohan, A., *et al.* SPECTER: Document-level Representation Learning using Citation-informed Transformers. In, *Proceedings of the 58th Annual Meeting of the Association for Computational Linguistics*. 2020. p. 2270-2282.

Conneau, A., *et al.* Supervised Learning of Universal Sentence Representations from Natural Language Inference Data. In, *Proceedings of the 2017 Conference on Empirical Methods in Natural Language Processing*. 2017. p. 670-680.

Dai, Z. and Callan, J. Context-aware term weighting for first stage passage retrieval. In, *Proceedings of the 43rd International ACM SIGIR conference on research and development in Information Retrieval*. 2020. p. 1533-1536.

Fiorini, N.*, et al.* Best Match: New relevance search for PubMed. *PLoS Biol* 2018;16(8):e2005343.

Fiorini, N.*, et al.* How user intelligence is improving PubMed. *Nat Biotechnol* 2018.

Gu, Y.*, et al.* Domain-specific language model pretraining for biomedical natural language processing. *ACM Transactions on Computing for Healthcare (HEALTH)* 2021;3(1):1-23.

Hirschman, L.*, et al.* Text mining for the biocuration workflow. *Database (Oxford)* 2012;2012:bas020.

Hofstätter, S.*, et al.* Efficiently teaching an effective dense retriever with balanced topic aware sampling. In, *Proceedings of the 44th International ACM SIGIR Conference on Research and Development in Information Retrieval*. 2021. p. 113-122.

Izacard, G.*, et al.* Towards unsupervised dense information retrieval with contrastive learning. *arXiv preprint arXiv:2112.09118* 2021.

Ji, Z.*, et al.* Survey of hallucination in natural language generation. *ACM Computing Surveys* 2023;55(12):1-38.

Johnson, J., Douze, M. and Jégou, H. Billion-scale similarity search with gpus. *IEEE Transactions on Big Data* 2019;7(3):535-547.

Karpukhin, V.*, et al.* Dense Passage Retrieval for Open-Domain Question Answering. In, *Proceedings of the 2020 Conference on Empirical Methods in Natural Language Processing (EMNLP)*. 2020. p. 6769-6781.

Khattab, O. and Zaharia, M. Colbert: Efficient and effective passage search via contextualized late interaction over bert. In, *Proceedings of the 43rd International ACM SIGIR conference on research and development in Information Retrieval*. 2020. p. 39-48.

Kingma, D.P. and Ba, J. Adam: A method for stochastic optimization. *arXiv preprint arXiv:1412.6980* 2014.

Le, Q. and Mikolov, T. Distributed representations of sentences and documents. In, *International conference on machine learning*. PMLR; 2014. p. 1188-1196.

Lee, J.*, et al.* BioBERT: a pre-trained biomedical language representation model for biomedical text mining. *Bioinformatics* 2020;36(4):1234-1240.

Lin, J. and Wilbur, W.J. PubMed related articles: a probabilistic topic-based model for content similarity. *BMC Bioinformatics* 2007;8:423.

Mialon, G.*, et al.* Augmented language models: a survey. *arXiv preprint arXiv:2302.07842* 2023.

Mikolov, T.*, et al.* Distributed representations of words and phrases and their compositionality. *Advances in neural information processing systems* 2013;26.

Neelakantan, A.*, et al.* Text and code embeddings by contrastive pre-training. *arXiv preprint arXiv:2201.10005* 2022.

Ni, J., *et al.* Large dual encoders are generalizable retrievers. *arXiv preprint arXiv:2112.07899* 2021.

Nogueira, R., Lin, J. and Epistemic, A. From doc2query to docTTTTTquery. *Online preprint* 2019;6.

Ostendorff, M., *et al.* Neighborhood contrastive learning for scientific document representations with citation embeddings. *arXiv preprint arXiv:2202.06671* 2022.

Pagliardini, M., Gupta, P. and Jaggi, M. Unsupervised Learning of Sentence Embeddings Using Compositional n-Gram Features. In, *Proceedings of the 2018 Conference of the North American Chapter of the Association for Computational Linguistics: Human Language Technologies, Volume 1 (Long Papers)*. 2018. p. 528-540.

Paszke, A., *et al.* Pytorch: An imperative style, high-performance deep learning library. *Advances in neural information processing systems* 2019;32.

Peters, M.E., *et al.* Deep Contextualized Word Representations. In, *North American Chapter of the Association for Computational Linguistics*. 2018.

Raffel, C., *et al.* Exploring the limits of transfer learning with a unified text-to-text transformer. *The Journal of Machine Learning Research* 2020;21(1):5485-5551.

Reimers, N. and Gurevych, I. Sentence-BERT: Sentence Embeddings using Siamese BERT-Networks. In, *Proceedings of the 2019 Conference on Empirical Methods in Natural Language Processing and the 9th International Joint Conference on Natural Language Processing (EMNLP-IJCNLP)*. 2019. p. 3982-3992.

Robertson, S. and Zaragoza, H. The probabilistic relevance framework: BM25 and beyond. *Foundations and Trends® in Information Retrieval* 2009;3(4):333-389.

Thakur, N., *et al.* BEIR: A heterogenous benchmark for zero-shot evaluation of information retrieval models. *arXiv preprint arXiv:2104.08663* 2021.

Tsatsaronis, G., *et al.* An overview of the BIOASQ large-scale biomedical semantic indexing and question answering competition. *BMC Bioinformatics* 2015;16:138.

Voorhees, E., *et al.* TREC-COVID: constructing a pandemic information retrieval test collection. In, *ACM SIGIR Forum*. ACM New York, NY, USA; 2021. p. 1-12.

Wadden, D., *et al.* Fact or Fiction: Verifying Scientific Claims. In, *Proceedings of the 2020 Conference on Empirical Methods in Natural Language Processing (EMNLP)*. 2020. p. 7534-7550.

Wang, L.L., *et al.* CORD-19: The COVID-19 Open Research Dataset. *ArXiv* 2020.

Wang, W., *et al.* Minilm: Deep self-attention distillation for task-agnostic compression of pre-trained transformers. *Advances in Neural Information Processing Systems* 2020;33:5776-5788.

Wolf, T., *et al.* Transformers: State-of-the-art natural language processing. In, *Proceedings of the 2020 conference on empirical methods in natural language processing: system demonstrations*. 2020. p. 38-45.

Wu, F., *et al.* Simplifying graph convolutional networks. In, *International conference on machine learning*. PMLR; 2019. p. 6861-6871.
Xiong, L., *et al.* Approximate nearest neighbor negative contrastive learning for dense text retrieval. *arXiv preprint arXiv:2007.00808* 2020.
Zhang, L., *et al.* A comparative evaluation of biomedical similar article recommendation. *J Biomed Inform* 2022;131:104106.
Zhang, Y., *et al.* BioWordVec, improving biomedical word embeddings with subword information and MeSH. *Sci Data* 2019;6(1):52.
Zhang, Y., *et al.* Multi-factor duplicate question detection in stack overflow. *Journal of Computer Science and Technology* 2015;30:981-997.
